# Mirvetuximab Soravtansine Induces Potent Cytotoxicity and Bystander Effect in Cisplatin-Resistant Germ Cell Tumor Cells

**DOI:** 10.3390/cells14040287

**Published:** 2025-02-15

**Authors:** Lucia Kucerova, Adriana Fekiacova, Natalia Udvorkova, Pavlina Malcharkova, Viktoria Blahova, Silvia Jochova, Katarina Kalavska, Zuzana Cierna, Michal Mego

**Affiliations:** 1 Translational Research Unit, 2nd Oncology Clinic of the Medical Faculty, Comenius University, and the National Cancer Institute, Klenova 1, 833 10 Bratislava, Slovakia; fekiacova27@uniba.sk (A.F.); natalia.udvorkova@savba.sk (N.U.); malcharkova1@uniba.sk (P.M.); viki.blahova@me.com (V.B.); katarina.kalavska@fmed.uniba.sk (K.K.); michal.mego@nou.sk (M.M.); 2 Cancer Research Institute, Biomedical Research Center, Slovak Academy of Sciences, 845 05 Bratislava, Slovakia; silvia.schmidtova@savba.sk; 3 Department of Genetics, Faculty of Natural Sciences, Comenius University, 841 04 Bratislava, Slovakia; 4 Medical Faculty, Comenius University, 813 72 Bratislava, Slovakia; 5 Department of Pathology, Faculty of Medicine, Comenius University, 811 08 Bratislava, Slovakia; ciernaz@gmail.com; 6 Department of Pathology, Faculty of Health Care and Social Work, University Hospital, 917 75 Trnava, Slovakia

**Keywords:** chemoresistance, cisplatin, antibody–drug conjugate, germ cell tumors, bystander effect, mirvetuximab soravtansine

## Abstract

Patients with treatment-refractory/relapsing germ cell tumors (GCTs) have a dismal prognosis due to a lack of any effective therapy. Moreover, the efficacy of newly approved targeted therapies remains unexplored for cisplatin-resistant GCTs. Previously, it was demonstrated that folate receptor α (FRα) is overexpressed in many tumor types and efficiently targeted by the antibody–drug conjugate (ADC) mirvetuximab soravtansine (MIRV) in cisplatin-resistant cancers. We hypothesized that FRα represents an attractive target for treating treatment-refractory GCTs. We determined the expression of the *FOLR1* gene in a broad range of GCT cell lines and tumor xenografts. We tested the antitumor efficacy of MIRV on cisplatin-resistant GCT cells in vitro and explored the ability of MIRV treatment to induce a bystander effect in the direct coculture of FRα-high and FRα-low cells. We found that the *FOLR1* gene has significantly higher expression in testicular GCTs (TGCTs) than in normal testicular tissue. *FOLR1* is highly expressed in the TCam2, JEG3, JAR, and NOY1 cell lines and their respective cisplatin-resistant variants. MIRV treatment induced apoptosis and a potent antiproliferative effect in cisplatin-resistant GCT cells in adherent and 3D spheroid cultures in vitro. A significant decrease in FRα-low 2102EP_R_NL cells was observed in the presence of FRα-high NOY1_R_SK in the presence of 12.5 nM MIRV, showing a potent bystander effect in the direct coculture. Immunohistochemical analysis confirmed significantly higher Folr1 protein expression in patients with TGCTs postchemotherapy than in chemo-naïve patients, as well as in patients with an unfavorable prognosis. In this study, we present data suggesting that the *FOLR1* gene is highly expressed in (T)GCT cells in vitro and in vivo, and anti-FRα-targeting therapies should be investigated as a treatment modality in a subset of patients with TGCTs. Moreover, MIRV induced significant antitumor and bystander effects, thus showing its potential in further preclinical exploration and drug repurposing for a salvage treatment regime in refractory (T)GCT disease.

## 1. Introduction

Current research into metastatic germ cell tumors (GCTs) focuses on finding novel biomarkers for effective salvage therapy for recurrent disease. Even though patients with GCTs with widespread metastatic dissemination can still be cured with a cisplatin (CPT)-based regimen, about 30% with metastatic disease at initial presentation (5–10% of all patients with GCTs) relapse or progress despite first-line treatment and, therefore, require efficient next-line therapy [1]. Cisplatin-resistant or refractory disease is linked to an extremely poor prognosis, highlighting the urgent need to identify new biomarkers and potential therapeutic targets in this context [2,3].

Recently, there has been major progress in the development of antibody–drug conjugates (ADCs) for treatment-refractory cancers, including modalities approved for use in solid cancers. A mirvetuximab soravtansine (MIRV) ADC, targeting folate receptor α (FRα)-expressing, cisplatin-resistant ovarian, fallopian tube, and peritoneal cancers, was approved in 2022 and has been recently reported to be effective and safe [4,5]. Nonmalignant tissues have limited FRα expression; however, it is expressed in 14–74% of non-small cell lung cancers, 72–100% of mesotheliomas, 20–50% of endometrial cancers, 35–68% of triple-negative breast cancers, and 76–89% of ovarian cancers. Thus, effective FRα-targeted therapeutic strategies have the potential to substantially improve the care of patients with an FRα+ malignant disease [6,7].

Four isoforms of the folate receptor (FR)—FRα, FRβ, FRγ, and FRδ—are distributed across different tissues and unidirectionally internalize folate into cells through the endocytosis of FRs. FRα, encoded by the *FOLR1* gene, binds folates with the highest affinity. FRα plays a vital role in embryonal development, as the knockout of the mouse equivalent of the *FOLR1* gene is embryonically lethal [8]. The crucial role of FRα in embryonal development was evidenced in a recent study by Balashova et al., who demonstrated that FRα is an essential factor in folding the neural tube in human-derived cells, as it aids in apico–basal pattern formation [9].

This protein is bound to the cellular surface through a glycosyl–phosphatidylinositol anchor. Recently, the localization of FRα on cellular membranes was demonstrated in a study by Jing et al., who showed that cancer cells displayed higher levels of FRα expression and had more dense distribution throughout membrane caveolae [10]. FRα expression decreases in healthy adult tissues, which could, in part, be attributed to a preference for other pathways in folate uptake, such as a protein-coupled folate transporter or a reduced folate carrier [11]. Interestingly, the possible engagement of FRα in chemoresistance was recently implied in gastric cancer. The overexpression of FRα led to higher levels of proliferating malignant cells in vitro; however, the depletion of this receptor did not completely kill malignant cells. Further evaluation of the role of FRα in gastric cancer cell survival revealed another contributor, an MDM2 protein, the involvement of which in the mechanisms of chemoresistance has already been suggested. The authors also showed that this intricate interplay between the MDM2 and FRα proteins is mediated by the protein prohibitin 2 PHB2 [12]. However, this putative association needs to be further assessed. Additionally, MDM2 has also been shown to be upregulated in chemo-resistant testicular cancer, and it could be interesting to assess its possible interaction with FRα in TGCTs regarding chemoresistance [13].

There is still an unmet medical need for safe and effective anticancer therapy for recurrent or refractory tumors. One strategy is to introduce ADCs designed to target FRα [14]. In clinical settings, the most advanced anti-FRα ADC is MIRV, which has already been approved by the Food and Drug Administration (FDA). MIRV is composed of an anti-FRα IgG1 monoclonal antibody that is chemically linked to a microtubule disruptor DM4 (payload). For linkage, a cleavable disulfide linker with lysine–amide coupling was used, and the average drug-to-antibody (DAR) ratio for MIRV was 3.5. Upon recognition by FRα, this conjugate can enter FRα+ cells, mediated by endocytosis. After lysosomal degradation, DM4 is released intracellularly and induces a G2/M cell cycle arrest, resulting in cell death [15]. MIRV was fully approved for treating platinum-resistant epithelial ovarian, fallopian tube, and peritoneal cancer patients who were previously treated systemically by the FDA in March 2024. [16]. Other FRα-directed ADCs are also showing promise and entering phase I clinical trials, such as LY4170156 (MBK-103) [17], AZD5335 (NCT05797168) [18], ZW191 (NCT06555744) [19], IMGN151 (NCT05527184), and Rinatabart sesutecan (PRO1184) (NCT05579366). Rinatabart sesutecan is an ADC that has been recently granted fast-track status by the FDA (https://www.onclive.com/view/fda-grants-fast-track-status-to-rinatabart-sesutecan-for-fr--expressing-ovarian-cancer, accessed on 10 February 2025). This ADC also very recently entered a phase III clinical trial for platinum-resistant ovarian cancer, which is estimated to start recruiting patients in December 2024 (NCT06619236). Luveltamab tazevibulin (STRO-002) also recently entered a phase II/III clinical trial (NCT05870748) for platinum-resistant ovarian cancer due to meaningful clinical activity, with an overall response rate (ORR) of 37.5% in a phase I trial [20]. So far, only a few of these ADCs have been tested on the JEG3 choriocarcinoma model in preclinical studies. IMGN151 has been shown to have antitumor effects on JEG3 cells, conferring an even higher efficacy than MIRV (IMGN853) (https://www.immunogen.com/wp-content/uploads/2020/06/IMGN151_AACR2020_PreClinical-Poster_vF-2.pdf, accessed on 6 February 2024). The activities of ZW191 (https://www.zymeworks.com/wp-content/uploads/2023/04/ZW191-poster-AACR23.pdf, accessed on 6 February 2024) and IKS01 were also preclinically tested on JEG-3 choriocarcinoma [21]. The significant antitumor activity and bystander effect of another novel FRα ADC, BAT8006, were recently reported in preclinical studies, whereby this ADC fully eliminated tumors in JEG3-derived mouse xenografts [22]. A very recent advancement was made in the field of drug conjugates, represented by ELU001, a nanoparticle drug pertaining to the novel group of C’Dot Drug Conjugates (CDCs). This conjugate was shown to be safe in an initial phase I clinical trial (NCT05001282), although this trial is currently on hold due to a lack of funding.

The potential of ADCs in treating recurrent/refractory GCTs has not been studied in detail, even though GCTs represent a suitable target for several ADC modalities [14]. In this study, we analyzed *FOLR1* gene expression in GCTs to determine whether GCTs represent treatment-refractory malignancy targetable by MIRV. We showed high FRα expression in multiple histological GCT subtypes. Importantly, we showed high *FOLR1* gene expression in cisplatin-resistant variants of GCT cell lines corresponding to their responsiveness to MIVR treatment in vitro. Our experiments also demonstrate the ability of MIRV to induce a potent bystander effect, thus targeting cells with low FRα expression and refractoriness to MIRV treatment. This suggests that MIRV can conjugate to tackle tumor heterogeneity and mixed histology in (T)GCTs.

## 2. Material and Methods

### 2.1. Cells and Material

This study employed a range of established human cell lines and their isogenic CPT-resistant variants, representative of all histological GCT subtypes.

Cell lines NCCIT_SK (ATCC^®^ CRL-2073™), NCCIT_NL (provided by Prof. Leendert H.J. Looijenga, Princess Máxima Center for Pediatric Oncology, Utrecht, The Netherlands), NCCIT_CZ (provided by Prof. Ludmila Boublikova, Charles University and University Hospital in Motol, Prague, Czech Republic), NEC8 (RCB0489, Cell Bank, RIKEN BioResource Research Center, Tsukuba, Ibaraki, Japan) NOY1 (ENG101, Kerafast, Shirley, MA, USA), JAR (ATCC^®^ HTB-144), SuSa (DSMZ ACC 747), and TCam2 (generously provided by Dr. Kitazawa from Ehime University Hospital, Shitsukawa, Japan) were maintained in RPMI 1640 medium (Sigma-Aldrich, Merck, Darmstadt, Germany) supplemented with 10% FBS (GIBCO^TM^, Life Technologies Corp., Grand Island, NY, USA), 1× Antibiotic Antimycotic Solution (Sigma-Aldrich, Merck), and 1× GlutaMAX^TM^ (GIBCO^TM^, Life Technologies Corp., Grand Island, NY, USA).

Cell lines TERA1, NTERA-2 [NT2] (ATCC^®^ CRL¬1973™), NT2_R_DE) (provided by Prof. Leendert H.J. Looijenga, Princess Máxima Center for Pediatric Oncology, Utrecht, the Netherlands), TERA2 _CZ (provided by Prof. Ludmila Boublikova, Charles University and University Hospital in Motol, Prague, Czech Republic), 2102Ep_NL (provided by Prof. Leendert H.J. Looijenga, Princess Máxima Center for Pediatric Oncology, Utrecht, the Netherlands), and JEG3 (ATCC^®^ HTB-36™) were cultivated in high-glucose (4.5 g/L) DMEM (Sigma-Aldrich, Merck) supplemented with 10% FBS (GIBCO^TM^, Life Technologies Corp., NY, USA), 1× Antibiotic Antimycotic Solution (Sigma-Aldrich, Merck), and 1× GlutaMAX^TM^ (GIBCO^TM^, Life Technologies Corp., Grand Island, NY, USA).

The NCR-G1 (JCRB1166, Japan) cell line was cultured in advanced DMEM/F12 supplemented with 10% FBS (GIBCO^®^ Invitrogen, Waltham, MA, USA), supplemented with 10% FBS (GIBCO^®^ Invitrogen), 1× Antibiotic Antimycotic Solution (Sigma-Aldrich, Merck), and 1× GlutaMAX^TM^ (GIBCO^TM^, Life Technologies Corp., NY, USA).

Some CPT-resistant variants were independently generated by three groups (Dr. Christoph Oing and Prof. Friedemann Honecker, University of Hamburg, Germany, referred to as DE or NL; Dr. Katarina Kalavska, Dr. Silvia Jochova-Schmidtova, and Prof. Michal Mego, Translational Research Unit, Faculty of Medicine, Comenius University, Bratislava, Slovakia, referred to as SK; and finally, Dr. Violeta Bakardjieva-Mihaylova and Prof. Ludmila Boublikova, Charles University and University Hospital in Motol, Prague, Czech Republic, referred to as CZ).

The CPT-resistant variants generated by the Translational Research Unit, Slovakia, were derived via the long-term propagation (6 months) of matched parental cells in sub-lethal concentrations of CPT (Hospira UK Ltd., Queensway Royal Leamington Spa, Tadworth, UK) without recovery time, as described previously [16]. The cells in the exponential growth phase were initially exposed to 0.165 µM of CPT. When the cells started to expand, the CPT concentration was gradually increased to 0.33 µM. Subsequently, de novo-derived CPT-resistant variants were continuously maintained in 0.33 µM of CPT in culture media.

Resistant clones, referred to as DE or NL, were generated from parental cells through repeated treatment with increasing sub-lethal concentrations of CPT with intermittent recovery time, as published in [23,24,25,26].

Finally, CPT-resistant cell lines, referred to as CZ, were derived from parental lines via culture with increasing CPT doses for 20 months. Establishing resistant cells started with a concentration of 0.1 μM CPT. When the lethality of cultivated cells reached 80%, the cells were left to recover over four passages without CPT. The final IC50 was 10 times higher than the original sensitive cells [27].

Other cells and cell lines—MDA-MB-231 (ATCC HTB-26^TM^), MCF10 (CRL-10317 ™), C33 (HTB-31™), Hs1.Tes (CRL-7002™) and HuFib (kindly provided by Dr. M. Matuskova, Cancer Research Institute BMC SAS, Bratislava, Slovakia)—were cultivated in high-glucose (4.5 g/L) DMEM (Sigma-Aldrich, Merck) supplemented with 10% FBS (GIBCO^TM^, Life Technologies Corp., NY, USA), 1× Antibiotic Antimycotic Solution (Sigma-Aldrich, Merck), and 1× GlutaMAX^TM^ (GIBCO^TM^, Life Technologies Corp., NY, USA). All cell lines were maintained at 37 °C under 5% CO_2_. Cell line identities were confirmed via short tandem repeat (STR) profiling (GENERI Biotech and Eurofins Genomics). Cell lines used in this work were screened regularly for mycoplasma (MycoAlert, LONZA, Basel, Switzerland).

The following compounds were used: mirvetuximab soravtansine solution (IMGN853; cat.no. HY-132258A, MedChem Express; Monmouth Junction, NJ, USA), MG132 (Cat. No. S2619, SelleckChem, Frankfurt am Main, Germany), DMSO (D8418, Sigma-Aldrich), and cisplatin (CPT; EBEWE Pharma GmbH, Nfg.KG, Unterach am Attersee, Austria).

### 2.2. Expression Analysis

Total RNA was isolated with the NucleoSpin^®^ RNA Mini Kit for RNA purification (Macherey-Nagel, Germany). The concentration and purity of the isolated RNA were determined using a NanoDrop™ 1000 Spectrophotometer (Thermo Fisher Scientific, Inc., Waltham, MA, USA). Alternatively, excised xenografts were homogenized using innuSPEED Lysis Tube A (Analytik Jena AG) and a rotor–stator homogenizer (Precellys^®^ 24 Touch, Bertin Technologies SAS Parc d’activités du Pas du Lac, Montigny-le-Bretonneux, France). Total RNA from xenografts was isolated using an innuPREP RNA Mini Kit 2.0 (Analytik Jena AG, Jena, Germany) and treated with NucleoSpin™ rDnase, according to the manufacturer’s instructions (Macherey-Nagel, Düren, Germany).

RNA was reverse-transcribed to cDNA using the GRiSP Xpert cDNA Synthesis Supermix (GRiSP Research Solutions, Portugal) or a RevertAid First Strand cDNA Synthesis Kit (Thermo Fisher Scientific, Inc., USA). The total RNA was calibrated for 2000 ng/reaction, and the reaction was prepared according to the manufacturer’s protocol to a total volume of 20 µL. The whole reaction was run on a T100 Thermal Cycler (Bio-Rad Laboratories, Inc., Hercules, CA, USA) at 37 °C for 15 min, 60 °C for 10 min, 95 °C for 1 min, and a final cooling at 4 °C. Alternatively, reverse transcription was performed at 65 °C for 5 min, 42 °C for 60 min, and 70 °C for 5 min using a RevertAid Kit. For gene expression analysis, a GoTaq^®^ qPCR Master Mix (Promega Corporation, Madison, WA, USA) was used according to the manufacturer’s instructions. The reaction mixture for the *FOLR1* gene was prepared to a total volume of 20 µL/reaction as follows: 10 µL of 2× GoTaq^®^ qPCR Master Mix, 1 µL of forward primer (10 pmol/µL), 1 µL of reverse primer (10 pmol/µL), 6 µL of Nuclease-Free Water, and 2 µL of cDNA template. The expression of the target gene was normalized to a housekeeping gene, hypoxanthine phosphoribosyltransferase 1 (*HPRT1*). The master mix for the *HPRT1* gene was prepared to a total volume of 20 µL/reaction as follows: 10 µL of 2× GoTaq^®^ qPCR Master Mix, 0.7 µL of forward primer (10 pmol/µL), 0.7 µL of reverse primer (10 pmol/µL), 6.6 µL of Nuclease-Free Water, and 2 µL of cDNA template. The sequences of the primers used in the reaction were *FOLR1* (forward) 5′-GCATTTCATCCAGGACACCT-3′, *FOLR1* (reverse) 5′-GGTGTAGGAGGTGCGACAAT-3′, HPRT1 (forward) 5′-GAACGTCTTGCTCGAGATGTGATG-3′, and HPRT1 (reverse) 5′-TGATGTAATCCAGCAGGTCAGCA-3′. cDNA was amplified with the CFX96™ Real-Time PCR Detection System (Bio-Rad Laboratories, Inc., USA) with the following thermal program: 95 °C for 2 min of initial denaturation, followed by 95 °C for 30 s, 60°C for 30 s, 72 °C for 30 s, 76 °C for 5 s, and 80 °C for 5 s for 40 cycles. This was followed by melting analysis from 70 °C to 95 °C, with an increment of 0.5 °C for 5 s at each step. Data were analyzed with CFX Maestro™ Software 1.0 (Bio-Rad Laboratories, Inc., USA). The relative level of expression was then calculated from the data using the standard 2^−ΔΔCt^ method, and the fold differences were compared with the selected reference cell line, SuSa, set to 1.

*FOLR1* gene expression in the xenografts was determined as described elsewhere in detail [28]. Briefly, NSG mice (age: 6–8 weeks; The Jackson Laboratory, Bar Harbor, ME, USA) were subcutaneously injected with a suspension of tumor cells and extracellular matrix (ECM Gel cat. no. E1270, Sigma-Aldrich) mixture diluted 1:1 with serum-free DMEM or RPMI medium. The xenografts were excised at the experimental endpoint, snap-frozen in liquid nitrogen, and stored at –80 °C until further processing. The project took place at the Animal Facility for Immunodeficient Mice of the Biomedical Research Center SAS Bratislava, operating under license no. SK UCH 02017. This project was approved by both the Institutional Ethics Committee of the Biomedical Research Center SAS Bratislava and the State Veterinary and Food Administration of the Slovak Republic, registration Nos. Ro 1030/18-221 and Ro 5862-3/2023-220. The project was conducted in accordance with Directive 2010/63/EU and Regulation 377/2012.

### 2.3. Viability Assay

Cells were plated at 5 × 10^3^ cells/100 µL of media per well in quadruplicates in a 96-well black-walled µClear plate (Greiner Bio-One, Kremsmünster, Austria) 24 h prior to treatment. MIRV was diluted in 100 µL of media and added to the cells at final concentrations ranging from 1.56 to 50 nM. Proteasome inhibitor MG132 at a concentration of 1µM was used as a control (maximum cytotoxicity) in each experiment.

The relative viability of the cells was determined using the CellTiter-Glo™ Luminescent Cell Viability Assay (Promega Corporation, USA) and evaluated by the GloMax^®^ Discover plate reader (Promega Corporation, USA) after 72 h of treatment according to the manufacturer’s recommendation. The relative viability value of the untreated controls was taken as 100%, and the data were calculated as the means ± SD of the quadruplicates.

### 2.4. Kinetic Impedance Assay for Cell Viability

For kinetic impedance-based measurements, the baseline for each plate was measured by adding 50 µL of each culture medium to CytoView MEA 96-well plates (Axion BioSystem). Cells were subsequently plated at 2 × 10^4^ cells/50 μL media per well and allowed to adhere overnight. The indicated treatment was added, and the experiments were performed in quadruplicates. Data are expressed as the means ± SDs of the impedance or % cytolysis. At the assay endpoint, the CellTiter-Glo™ Luminescent Cell Viability Assay (Promega Corporation, USA) was used to determine endpoint viability in the same plate, and the relative viability was evaluated using the GloMax^®^ Discover System plate reader (Promega Corporation, USA). The relative viability value of the untreated controls was taken as 100%.

### 2.5. Three-Dimensional Culture and Spheroid Viability Assays

Cells were seeded in a Nunclon™ Sphera U-Shaped-Bottom 96-Well Microplate (Thermo Scientific, Inc.; USA) at a seeding density of 5 × 10^3^ cells per well in 100 µL of adequate media in quadruplicates. The microplate was cultivated for 3 days to enable spheroid formation. After cultivation, the microplate was treated with MIRV diluted to 100 µL of media per well in a serial dilution (1.56–50 nM). Following treatment, spheroids were incubated for an additional 4 days. At the assay endpoint, the CellTiter-Glo^®^ 3D Cell Viability Assay (Promega Corporation, USA) was used according to the manufacturer’s instructions. The data are expressed as the mean ± SD of the quadruplicates.

### 2.6. IncuCyte^®^ Live-Cell Analysis of Cell Proliferation

Before live-cell analysis, cells were transduced to express either the mKate2 protein or the tagGFP2 protein. Cells were seeded 24 h prior to transduction to reach 25–35% confluency at the time of infection. Incucyte^®^ Nuclight Red (NLR) Lentivirus (puro) reagent (Cat. No. 4625, Essen BioScience Ltd.—A Sartorius Company, Welwyn Garden City, UK) or Incucyte^®^ Nuclight Green (NLG) Lentivirus (puro) reagent (Cat. No. 4624, Essen BioScience Ltd.—A Sartorius Company, UK) was diluted in a medium containing 8 μg/mL Polybrene^®^ and added at MOI = 3. Cells were transduced at 37 °C in 5% CO_2_ for 24 h. The transduction medium was removed and refreshed for the following 48 h culture. Cells were harvested, expanded, and frozen. For stable expression, we performed antibiotic selection, adding 1 μg/mL of puromycin to complete culture medium. The medium was replaced every 48–72 h, and the expression of the nuclear fluorescent label was monitored with Incucyte^®^ Live-Cell Analysis System IncuCyte^®^ZOOM (Essen BioScience Ltd., Welwyn Garden City, UK).

Quadruplicates of the nuclear-labeled cells were plated at a seeding density of 5 × 10^3^ cells/100 µL media per well in a 96-well black-walled plate with a µClear flat bottom (Greiner Bio-One, Austria). Cells were treated with indicated concentrations of MIRV or CPT for 24 h after seeding. Incucyte^®^ Caspase-3/7 Dye for Apoptosis (1000×, Sartorius, Gottingen, Germany) was added to selected wells according to the manufacturer’s recommendation. Cell viability was monitored using the Incucyte^®^ZOOM system, and images were taken at a 10X objective in phase contrast to monitor culture confluence and fluorescence intensity every 2–3 h. Data were analyzed using IncuCyte^®^ZOOM, version 2016A. Data are expressed as the mean ± SD of the fluorescent cell count per image. At the assay endpoint, the CellTiter-Glo™ Luminescent Cell Viability Assay (Promega Corporation, USA) was used as described above.

### 2.7. IncuCyte^®^ Live-Cell Analysis of Bystander Effect

To analyze the bystander effect, cells were seeded at a density of 1 × 10^4^ cells/100 µL of media per well in quadruplicates in a 96-well black-walled plate with a µClear flat bottom (Greiner Bio-One, Austria). The microplate contained selected cell lines with *FOLR1*-higher expression (NLR-NOY-1_R_SK or NLR-JEG3_R_SK), mixed with *FOLR1*-lower-expressing NLG-2102Ep_R_NL, which were plated alone or in a mixed coculture. After a 24 h incubation period, the cells were treated with the indicated concentrations of MIRV. Cell viability was monitored using the Incucyte^®^ZOOM system as described above. At the assay endpoint, 10X RIPA buffer (Cell Signaling Technology, Inc., Danvers, MA, USA) was used to induce cell lysis in the cells according to the manufacturer’s protocol. After cell lysis, the relative green fluorescence was determined using the GloMax^®^ Discover System plate reader (Promega Corporation, USA). Data were expressed as the mean of the technical quadruplicates ± SD. The value of the untreated cells was set to 100%.

### 2.8. Immunohistochemical Analysis

This study included 64 patients with newly diagnosed GCTs treated with systemic therapy at the National Cancer Institute and the St. Elizabeth Cancer Institute (Bratislava, Slovakia), for whom paraffin-embedded tumor tissue samples were available in a tissue biobank. All patients were treated with first-line cisplatin-based therapy, and there were no primary cisplatin-refractory patients. The aforementioned institutes are national comprehensive cancer centers and referral centers for GCTs in Slovakia. Therefore, the patient population in the present study and the distribution of stages and histology do not represent this distribution in the general GCT population of Slovakia. Patients with concurrent malignancy other than nonmelanoma skin cancer in the previous five years were excluded. Patient/tumor characteristics, delivered systemic therapy, and treatment outcomes were collected from all patients. This study received approval from the Institutional Review Board, and a patient consent waiver was obtained. The classification of GCTs was carried out according to the criteria established by the World Health Organization. Analysis was performed as previously described in detail [28].

Slides were incubated in Tris/EDTA retrieval solution (pH 9.0) at 97 °C for 20 min using the automated water bath heating process in Dako PT Link (Dako, Agilent Technologies, Inc., Santa Clara, CA, USA) for tissue epitope demasking. The slides were pre-treated with hydrogen peroxide for 5 min to block endogenous peroxidase activity. The slides were subsequently incubated for 30 min at room temperature with the primary monoclonal mouse antibody against *FOLR1* (Proteintech, clone no. 2B4B7, cat. no. 60307-1-Ig), which was diluted 1:100 in Dako REAL antibody diluent (Dako; Agilent Technologies, Inc.). To improve the sensitivity of the reaction, the slides were incubated with mouse (LINKER) for 15 min. Immunostaining was carried out using an anti-mouse/anti-rabbit immuno-peroxidase polymer (EnVision FLEX/HRP; Dako; Agilent Technologies, Inc.) for 20 min at room temperature, according to the manufacturer’s instructions. To visualize the reaction, a diaminobenzidine substrate–chromogen solution (DAB; Dako; Agilent Technologies, Inc.) was applied for 10 min. Finally, the slides were counterstained with hematoxylin for 5 min and mounted for analysis. *FOLR1* positivity in human kidney tissue was used as a positive control, and the same tissue omitting the primary antibody served as a negative control.

FOLR1-stained tissue microarray (TMA) slides were scanned using the microscope slide scanner Aperio AT2 (Leica) at 20× magnification. The analysis was performed using the bioimage analysis software QuPath (v0.5.1) on an Acer Nitro 5 (2.9 GHz, 14-Core Intel Core i9-12900H, 32-GB RAM) [29]. The slides were uploaded to the QuPath software as Brightfield (H-DAB) images, and TMA dearraying was performed. The cores were manually examined and excluded from the analysis if deemed unsuitable due to insufficient tumor tissue or poor quality. To improve stain separation, deconvolution was conducted using the “‘Estimate stain vectors” command. Cells were identified using the cell detection function based on the optical density of nuclear hematoxylin staining. Due to significant heterogeneity among the tissue cores within each project, the cell detection parameters—sigma, threshold, and area—were varied to optimize cell identification. The background radius was set to 0 to ensure accurate cell detection in dense cellular areas and to avoid detection variability across tiles. Since the DAB signal was localized to the cytoplasm, the cell expansion was reduced to 2 μm to minimize the risk of making measurements within neighboring cells. Representative areas were annotated by a pathologist (ZC), and a random trees classifier was built to distinguish tumor cells from all other detections. The intensity thresholds for the cytoplasm DAB optical density mean were set to 0.2, 0.4, and 0.6 to classify cells into negative, weak, moderate, or strong staining groups. In addition to the manual examination prior to the analysis, a subset of tissue cores was excluded from the study due to poor cell segmentation caused by obscured cellular morphology and the inability to develop a reliable object classifier.

### 2.9. Statistical Analysis

Due to the non-normal distribution of HS values, as indicated by the Shapiro–Wilk normality test, nonparametric tests were employed for further analysis. The Mann–Whitney U test was used to assess differences in the distribution of *FOLR1* levels between two groups of patients. For comparisons between more groups, the Kruskal–Wallis test was performed. In cases where the *FOLR1* level was categorized as “low” or “high” based on the specified criteria, Fisher’s exact test or the χ^2^ test was used for analysis. *FOLR1* was dichotomized as “high” or “low” based on the median.

The median follow-up period was determined by calculating the median observation time for all patients, including those still alive at their last follow-up. Progression-free survival (PFS) was calculated from the date of orchiectomy or tumor biopsy to the date of disease progression, death, or last follow-up. Overall survival (OS) was calculated from the date of orchiectomy or tumor biopsy to the date of death or last follow-up. The Kaplan–Meier product limit method was used to estimate PFS and OS, and a log-rank test was employed to compare survival between groups. Statistical analysis was conducted using the NCSS 2019 software version 19.0.9 (NCSS 2019 Statistical Software, NCSS, LLC, Kaysville, UT, USA). A *p* < 0.05 indicated a statistically significant difference.

## 3. Results

**Figure 1:**
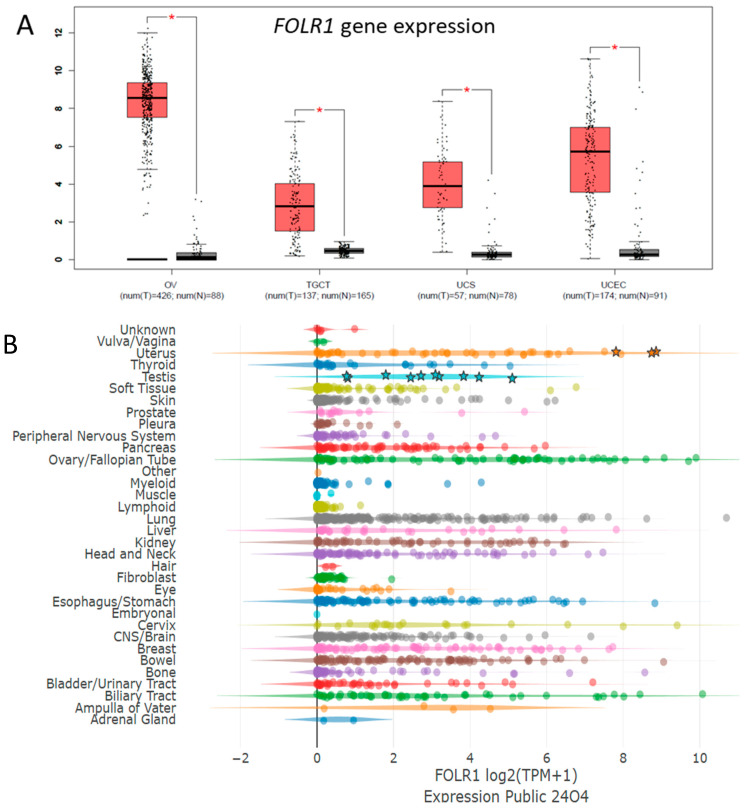

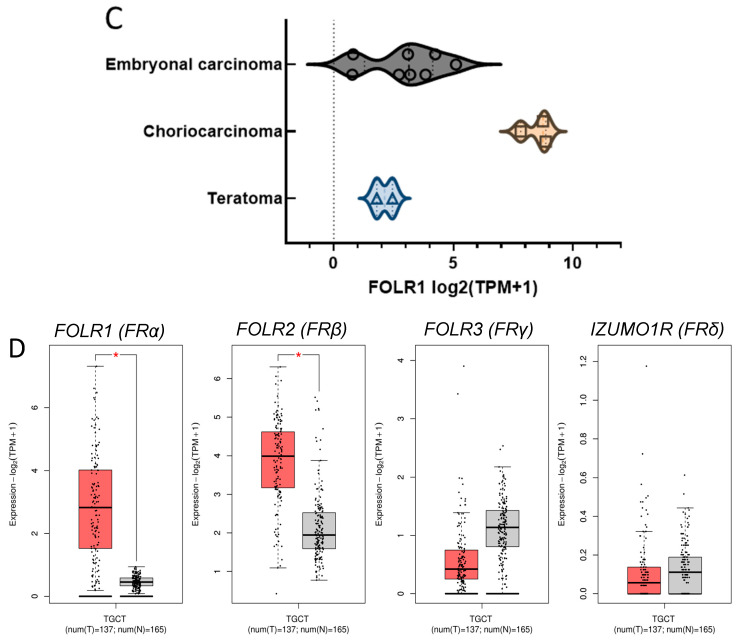
*FOLR1* gene expression in TGCTs. (**A**) The *FOLR1* gene is highly expressed and significantly upregulated in TGCTs (red) compared with healthy testicular tissue (gray), as well as in other tumor types, such as ovarian cancer (OV), uterine carcinosarcoma (UCS), and uterine corpus endometrial carcinoma (UCEC). (**B**) *FOLR1* expression in the cancer cell lines according to the tissue of origin available from the Cancer Cell Line Encyclopedia (CCLE) public database. Asterisks indicate *FOLR1* expression in the TGCT and ChC cell lines; each color illustrates different tissue lineages. (**C**) Detailed image depicting *FOLR1* expression in (T)GCT cell lines 1156QE8, 1618K, 1777NRPMET, 833KE, NCCIT, TERA1, TERA2, NTERA2CLD1 (EC, dark gray); GCT27 and SUSA (TC, blue); and JAR, JEG3, and T3M3 (ChC, light brown) according to the CCLE. (**D**) *FOLR2* expression is also significantly upregulated in TGCT tissue (red) compared with normal tissue (gray), but there is no significant difference in the expression of *FOLR3 or IZUMO1R* (FRδ). * *p* ≤ 0.05.

**Figure 2:**
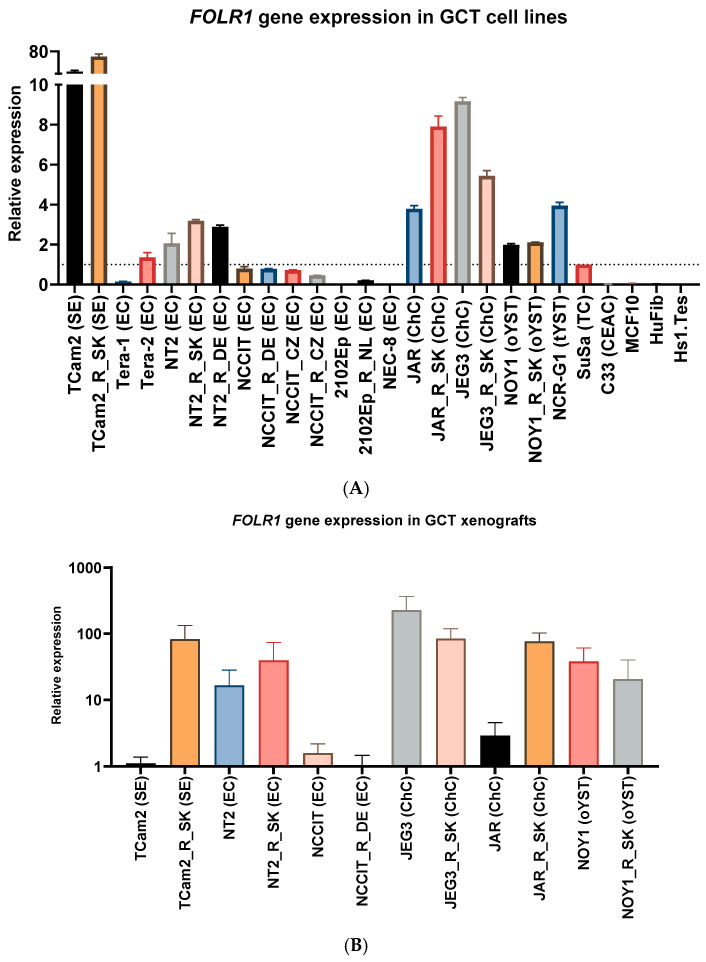
*FOLR1* gene expression in cisplatin-resistant GCT cell lines and xenografts. (**A**) Quantitative RT-PCR was used to determine the relative expression level of the *FOLR1* gene in a panel of GCT cell lines. The panel includes established cell lines inherently resistant to CPT and pairs of sensitive and derived cisplatin-resistant variants of GCTs of all histological subtypes. C33 cervical adenocarcinoma cell lines and MCF10 mammary epithelial cells were used to confirm low/absent expression in these cells, as expected according to the CCLE. Normal human testicular fibroblast Hs1.Tes and human foreskin fibroblast HuFib were used to confirm very low *FOLR1* gene expression in nonmalignant cells. The data are expressed as fold changes in expression, where the expression in SuSa (TC) cells was taken as a reference. (**B**) Quantitative RT-PCR was used to determine the relative expression level of the *FOLR1* gene in xenografts derived from selected GCT cell lines in vivo. High *FOLR1* gene expression was confirmed in xenografts derived from seminoma, choriocarcinoma, and yolk sac tumor parental cells and their resistant variants.

**Figure 3:**
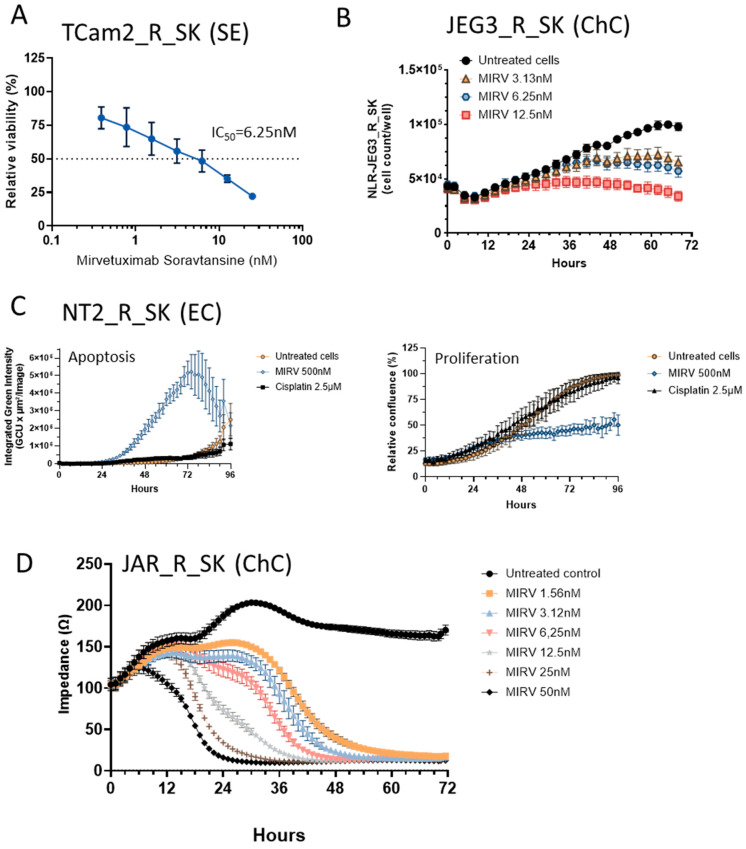
Effect of mirvetuximab soravtansine (MIRV) on cisplatin-resistant GCT cells. (**A**) TCam2_R_SK cisplatin-resistant seminoma cells were treated with increasing doses of MIRV in vitro. Viability was determined using a luminescent viability assay, illustrating the unique sensitivity of the seminoma cells to MIRV treatment at nanomolar concentrations. (**B**) Live-cell imaging assays confirmed a significant decrease in the cell number of NLR-JEG3_R_SK choriocarcinoma cells upon treatment with a 3.13nM dose of MIRV. (**C**) Induction of apoptosis via MIRV in cisplatin-resistant NT2_R_SK cells. NT2_R_SK (EC) cells were treated with 500 nM of MIRV or 2.5 µM of CPT in vitro. Apoptosis induction was kinetically monitored using a green fluorescent signal from Incucyte^®^ Caspase-3/7 Dye for apoptosis in a live-cell imaging system. A significant increase in green signal and apoptosis induction was observed starting 36 h post-treatment (left panel). Relative confluence in the same experiment exhibited a significant decrease starting 48 h post-treatment, corresponding to apoptosis induction and the antiproliferative effect of MIRV (right panel). No significant changes in fluorescence or relative confluence were observed upon treatment with 2.5 µM of CPT corresponding to the cisplatin resistance of the target NT2_R_SK cells. (**D**) MIRV induced a decrease in cell impedance, as a readout of cytolysis was determined in a kinetic-based impedance assay. JAR_R_SK (ChC) cells were plated onto CytoView plates, and after 24 h, various concentrations of MIRV were added. Dose–response and a decrease in impedance were monitored for 96 h. MIRV at a concentration of 1.56 nM induces complete cell detachment in vitro.

**Figure 4:**
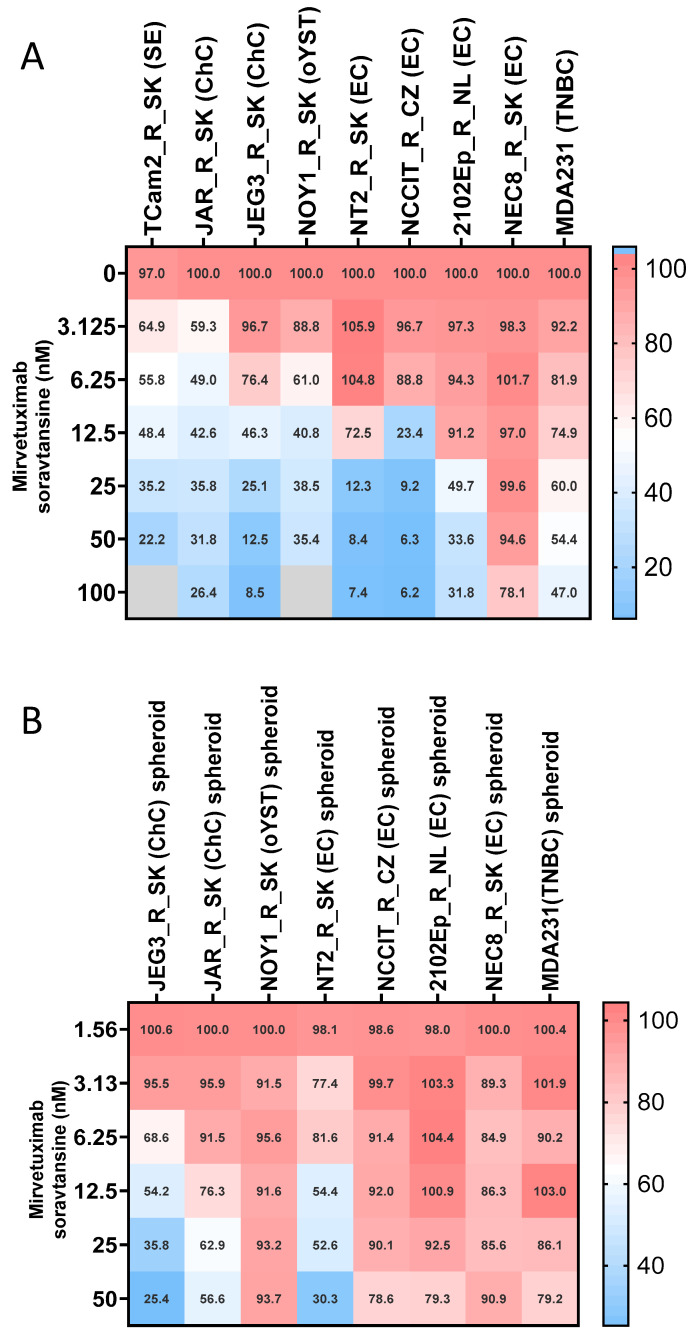
Antiproliferative effect of MIRV in adherent and spheroid cultures. (**A**) GCT cells were treated with MIRV, and viability was determined using a luminescent assay. GCT cell lines are aligned according to their sensitivity to the MIRV treatment in vitro, showing the highest sensitivity of TCam2_R_SK (SE), JEG3_R_SK (ChC), JAR_R_SK (ChC), and NOY1_R_SK (oYST). Cisplatin-resistant MDA-MB-231 triple-negative breast cancer (TNBC) cells were included to compare the cytotoxic effect (*FOLR1* expression, 2.55 in CCLE). (**B**) GCT cells were grown as spheroids in 3D culture conditions and treated with increasing concentrations of MIRV. Subsequently, the spheroid viability was determined using an endpoint 3D luminescent assay. JEG3_R_SK (ChC), JAR_R_SK (ChC), and NT2_R_SK (EC) cells retained their sensitivity to MIRV treatment in 3D culture conditions. Mean viability values are shown for each treatment condition; red depicts values above IC50, white corresponds to IC50, and blue illustrates the lowest viability values.

**Figure 5:**
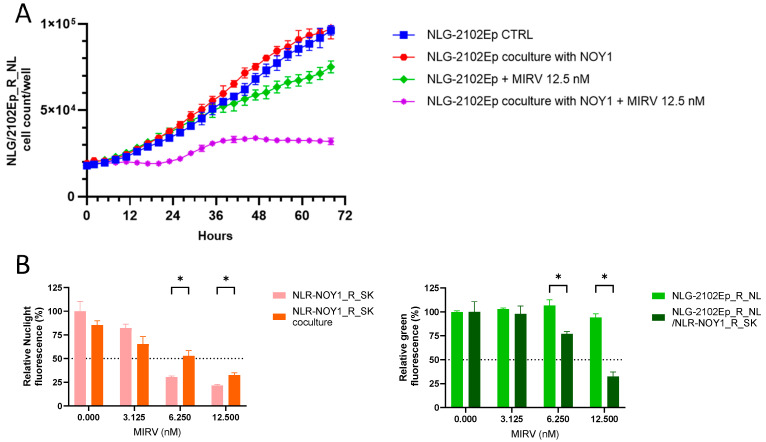
Bystander effect induced by MIRV into GCT cells. (**A**) Green fluorescent NLG-2102Ep_R_NL cells were cultured, whether alone or in coculture, with red fluorescent NLR-NOY1_R_SK cells with or without 12.5 nM MIRV. The cell number of the green 2102Ep_R_NL cells was evaluated using live-cell imaging in a kinetic proliferation assay. There was no difference between the proliferation of the *FOLR1*-refractory 2102Ep_R_NL cells in the coculture and the more sensitive NOY1_R_SK cells. MIRV induced only the limited inhibition of proliferation in 2102Ep_R_NL cells treated alone; however, in the coculture with MIRV-responsive NOY1_R_SK cells, there was substantial cell proliferation inhibition. (**B**) The endpoint evaluation of the relative fluorescence demonstrated a potent bystander cytotoxicity effect induced by MIRV in the presence of more sensitive NOY1_R_SK cells in the 2102Ep_R_NL refractory target. As an indication of viability, relative fluorescence is significantly lower in the donor cells (left panel) and significantly higher in the target cells with low *FOLR1* gene expression and low sensitivity to MIRV (right panel) when cocultured together in the presence of MIRV. * *p*-value ≤ 0.05.

First, we focused on evaluating *FOLR1* gene expression in tumor tissue, GCTs, and their cisplatin-resistant variants. We found that *FOLR1* is expressed in TGCTs, with significantly higher levels in the tumor samples than in normal testes tissue by searching in publicly available research databases. The *FOLR1* expression level is similar and follows the same pattern as in ovarian and uterine cancers (Figure 1A), according to the GEPIA database [30]. These cancers were already approved as indications for FRα-targeting therapy with mirvetuximab soravtansine (MIRV). Expression data in the Cancer Cell Line Encyclopedia (CCLE) database indicate high *FOLR1* gene expression in the majority of cell lines of all lineages, including those of testicular origin (Figure 1B) [31]. More specifically, the expression was confirmed in the embryonal carcinoma (EC) cell lines 1777NRPMET, 1618K, NCCIT, NTERA2CLD1, and TERA2 and teratocarcinoma (TC) cell line SuSa (Figure 1C). The GEPIA data also indicate significantly higher *FOLR2* gene expression in TGCT samples than in normal tissue, but no difference was shown for *FOL3* gene expression (Figure 1D). To unravel the expression pattern in other model cell lines and their cisplatin-resistant variants, we performed expression analysis on a broad panel of GCT cell lines and their cisplatin-resistant variants (Figure 2A). We used the TC cell line SuSa as a reference, as it is reported (in the CCLE and Human Protein Atlas datasets (available at www.proteinatlas.org accessed on 20 January 2025) to express the *FOLR1* gene. The highest expression was observed in the seminoma (SE) cells TCam2 and TCam2_R_SK. We also confirmed expression in choriocarcinoma cells (ChCs) JEG3, JAR, and their resistant variants; yolk sac tumor (YST) cells NOY1 and NCR-G1; and in NT2 and cisplatin-resistant EC cells. Moreover, when these cells were injected into immunodeficient animals, and the RNA was isolated from the growing xenografts, our results confirmed high FRα expression in vivo (Figure 2B). Thus, we decided to next evaluate the efficacy of the ADC targeting FRα via MIRV in cytotoxicity assays in vitro, aiming to treat cisplatin-resistant disease. Our data suggested that the target was highly expressed in the resistant cells; thus, we focused on these in our cytotoxicity assays. TCam2_R_SK (SE) exhibited an IC_50_ for MIRV at a 6.25 nM concentration, illustrating its high efficacy in this model (Figure 3A). Moreover, very low concentrations of MIRV also substantially decreased the cell numbers in JEG3_R_SK (ChC) cells in a kinetic live-cell assay (Figure 3B). Next, we demonstrated that the potent cytotoxicity effect of MIRV is mediated by apoptosis induction in a kinetic apoptosis assay. NT2_R_SK (EC) cells treated with MIRV exhibited significant increases in their green signal, mediated by Casp3/7 activation, which started at 36 h post-treatment (Figure 3C, left). Apoptosis activation was followed by a significant decrease in cell confluence, which is indicative of the strong antiproliferative effect of MIRV visible at 48 h and later after the start of treatment (Figure 3C, right). As summarized in Figure 4A, these cell lines, along with JAR_R_SK (ChC), NOY1_R_SK (oYST), and NCCIT_R_CZ (EC), were the most sensitive to MIRV treatment in adherent cultures. The sensitivity of 2102Ep_R_NL is similar to MDA-MB-231 breast cancer cells, with an IC_50_ of 25nM for MIRV. Next, we switched the GCT cells to spheroid culture conditions in ultra-low attachment plates with U-bottoms. The TCam2_R_SK cells did not form spheroids, and they did not proliferate under these conditions. The other cell lines were treated with various concentrations of MIRV, and high sensitivity was confirmed for JEG3_R_SK, JAR-R-SK, and NT2_R_SK cells at IC_50_ ≤ 25 nM. Other cell lines did not exhibit inhibition in spheroid proliferation at low MIRV concentrations (Figure 4B). Importantly, bystander cytotoxicity was described for ADC therapeutics [22,32]. Specifically, if there was any bystander effect induced by MIRV, we cocultured fluorescently labeled cells to follow the viability of each cell type in the direct coculture. In a combination of highly sensitive NOY1_R_SK (red fluorescent, NLR) and MIRV, the viability of the more refractory 2102Ep_R_NL (green fluorescent, NLG) was significantly reduced compared with the MIRV treatment alone (Figure 5A). This is due to a potent bystander effect, confirmed by a significant decrease in endpoint relative green fluorescence (Figure 5B).

**Figure 6:**
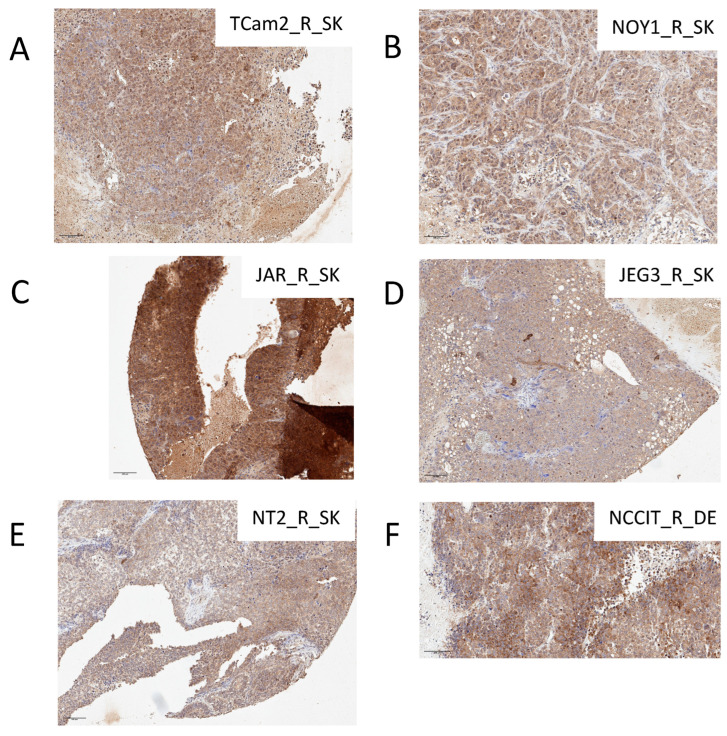
Immunohistochemical staining of *FOLR1* protein in cisplatin-resistant GCT cell line xenografts TCam2_R_SK (**A**), NOY1_R-SK (**B**), JAR_R_SK (**C**), JEG3_R_SK (**D**), NT2_R_SK (**E**), and NCCIT_R_SK (**F**). Original magnification: ×200; scale bar: 100 μm. Visualization with 3,3′-diaminobenzidine.

Next, we analyzed the *FOLR1* protein in tumor xenografts and patient tumor tissues. We confirmed high expression and IHC positivity in representative xenografts derived from the cisplatin-resistant cell line variants (Figure 6). The patient characteristics are summarized in Table 1. The median age was 31 years (18–61 years). The patient group consisted of 64 people, including 17 (26.6%) with pure seminomas and 47 (73.4%) with non-seminomas. Eleven patients had embryonal carcinomas (EC), zero had yolk sac tumors (YST), and one had choriocarcinoma (ChC) and two immature teratomas, while 33 tumors were mixed GCTs. Most patients (48, 75%) had a good prognosis according to the IGGCCG risk group. A favorable response to treatment was achieved in 92.2% of patients, while 6.3% had an unfavorable response. Moreover, the study included five patients with viable germ cell tumors in retroperitoneal lymph nodes obtained during retroperitoneal lymph node dissection.

**Figure 7:**
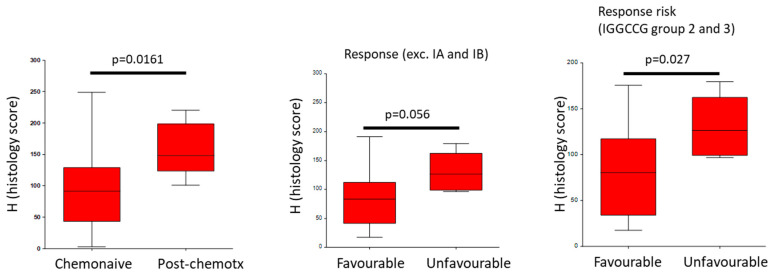
Immunohistochemical analysis of *FOLR1* in TGCT patient samples. The *FOLR1* level in postchemotherapy-viable tumors was significantly higher than in chemotherapy-naïve TGCTs. There was a significant association between *FOLR1* levels in tumor tissues and unfavorable treatment responses when stages IA and IB were excluded or in patients with intermediate/high risk according to their IGGCCG scores.

The mean ± standard deviation (SD) of the *FOLR1* level detected using IHC in the GCTs was 94.1 ± 56.8. No significant difference was observed between seminoma (97.5 ± 56.1), ChC (97.1 ± 60.4), embryonal carcinoma (69.5 ± 51.4), YST (104.6 ± 77.6), or teratoma (100.6 ± 35.7) (*p* = 0.2986). There was a significant association between the *FOLR1* level in tumor tissue and unfavorable treatment responses when stages IA and IB were excluded or when patients with intermediate/high risk were considered according to their International Germ Cell Collaborative Group (IGGCCG) scores (*p* = 0.05 and *p* = 0.03, respectively) (FIG.7). Importantly, *FOLR1* levels in postchemotherapy-viable tumors were significantly higher than in chemotherapy-naïve testicular germ cell tumors (mean ± SD, 158.6 ± 44.0 vs. 94.1 ± 56.8, *p* = 0.01608) (Figure 7).

In the median follow-up of 36.9 months (0.2–183.3 months), 16 (25.0%) patients experienced disease progression, and 7 (10.9%) died. There was no difference in PFS or OS between patients with “low” vs. “high” *FOLR1* levels (HR = 0.99, 95% CI 0.37–2.66, *p* = 0.98 for PFS and HR = 0.79, 95% CI 0.18–3.46, *p* = 0.75). Subgroup analysis revealed a lack of prognostic value for *FOLR1* in patients with testicular GCTs (Supplementary Table S1).

Taken together, we have demonstrated the substantial efficacy of MIRV in treating refractory GTC cells with high *FOLR1* gene expression. Importantly, our data show a potent bystander effect that improves treatment efficacy in tumors with mixed histology and the heterogeneous expression of the targeted antigen. In conclusion, our data provide evidence for extending the indication for MIRV in a clinical setting as a next-line treatment for patients with FRα-positive recurrent/refractory TGCTs.

## 4. Discussion

GCTs are the most common type of cancer in adolescent boys and young adult men, with an incidence of 5.8–11.3 per 100,000 in Western countries and 1 or 2 per 100,000 in Japan [33]. There is a small proportion of patients with TGCTs who are not cured with first-line, conventional dose, or high-dose salvage chemotherapy. Nearly all of them are expected to die from TGCTs; these deaths result in the greatest number of per-patient life years lost of any adult malignancy. (T)GCTs represent one of the few tumor types that have not yet benefited from targeted therapies. Several studies have evaluated the effect of antiangiogenic agents on patients with relapsed or refractory TGCTs with minimal efficacy [34]. Only anecdotal responses have been observed with immune checkpoint inhibitors among a few patients with relapsed or refractory TGCTs [35]. Additional ongoing studies have included a phase I trial of the hypomethylating agent guadecitabine combined with cisplatin, which showed some activity, and two phase II studies of second-generation taxane cabazitaxel [36]. There is an urgent need for a new therapy for relapsed and refractory TGCTs [33].

Recent progress in ADC development has shown promise in tackling drug resistance, tumor heterogeneity, and treatment-related adverse effects. ADCs combine three structural components: the antibody, the cytotoxic payload, and the chemical linker that connects them [4]. Intensive research analyzing specific tumor-associated surface proteins will soon provide many more novel therapeutics [37,38]. Novel ADC-based therapies have been investigated to a very limited extent for the treatment of relapsed/refractory TGCTs, even though there are multiple options to target TGCT surfaceomes [14]. So far, the ADC brentuximab vedotin—which targets CD30, an antigen expressed by embryonal carcinoma—has not exhibited clinically meaningful single-agent activity in patients with refractory GCTs in a phase II clinical trial [39,40]. The oncofetal antigen Claudin 6 (CLDN6) is highly expressed in TGCTs; thus, the efficacy of CLDN6-targeting ADC has been tested, but only with limited efficacy [41,42]. However, patients with GCTs treated with CLDN6-specific CAR-T cells plus amplifying RNA vaccine have exhibited a promising overall response rate of 57% (four out of seven) [43].

The potential to inhibit FRα in cancer therapeutics has been demonstrated using various moieties for multiple cancers (as recently reviewed in [6]). Upon the discovery of the folate pathway, the therapeutic approach was to inhibit the thymidylate synthase (TS) enzyme. The first antitumor agent, 5-fluorouracil (5-FU), was developed as an inhibitor of TS. First synthesized in 1957 [44], this inhibitor is still actively used as a first-line cancer therapeutic, with various advancements being made in its delivery to cells, such as the use of various nanosized vehicles, like nanohydrogels [45], exosomes [46], and folate-engineered nanoparticles [47]. Recent developments in TS inhibition also include a small-molecule inhibitor, CT900 (ONX-0801 or BCG945), which was tested in a phase I clinical trial (NCT02360345). This moiety enters the cell through FRα-mediated endocytosis upon binding to the folate receptor, reflecting the higher efficacy of CT900 seen in patients with higher FRα expression [48].

In 1947, another pioneering drug agent was developed as a folic acid pathway inhibitor: methotrexate, also known as amethopterin [49]. It inhibits the activity of the enzyme dihydrofolate reductase (DHFR) [50]. Interestingly, in TGCT cell lines, treatment with methotrexate leads to a resistant phenotype after reaching a certain concentration, where a seemingly set number of TGCT cells cannot be killed, experiencing a plateauing therapeutic response [39]. An advancement in methotrexate was introduced with the development of a new-generation antifolate, pemetrexed, which can inhibit more than one component of the folate pathway, including TS, DHFR, glycinamide ribonucleotide formyltransferase (GARFT), and aminoimidazole carboxamide ribonucleotide formyltransferase (AICARFT) [51]. Another class of drugs used to target the folate pathway are small-molecule drug conjugates (SMDCs), which include vintafolide and a novel EC2629 conjugate [52]. The immunotherapeutic approach has also been explored regarding FRα, which involved the monoclonal antibody farletuzumab (MORAb-003). This showed meaningful preclinical activity. However, a phase III clinical trial with platinum-resistant ovarian cancer patients did not demonstrate superior antitumor activity for this agent [53,54]. Methotrexate combined with paclitaxel, ifosfamide, and cisplatin was evaluated in poor-risk, non-seminomatous germ cell tumors in a phase II trial, showing an overall favorable response of 76.6%; however, the contribution of adding methotrexate to the cisplatin–chemotherapy backbone cannot be assessed based on this study [55].

There is limited information regarding the expression of the *FOLR1* gene in TGCTs and its association with clinicopathological characteristics. In seminoma tissue, the encoded receptor FRα is expressed in lower amounts than in nonmalignant testicular tissue [56]. When compared with the expression of KB cells (originally thought to be derived from an epidermal carcinoma of the mouth but subsequently found to have been established via HeLa cell contamination), the JEG-3 ChC cell line exhibited a lower but still substantial level of FRα expression [57]. Lower FRα expression in JEG3 cells was also demonstrated via immunostaining in a study by Simanjuntak et al., where JEG3 showed higher levels of the folate transporter instead of the folate receptor [58]. More recently, the JEG3 and JAR cell lines were evaluated regarding methotrexate uptake, along with their respective expressions of FRα. This study showed that these cell lines express FRα in higher amounts, especially compared with other cellular transporters for folic acid [59]. This finding is consistent with a previous study, where JEG3 and JAR cells were shown to overexpress *FOLR1* at the mRNA level [60].

Taken together, the published information regarding *FOLR1* expression in TGCTs is still limited (the CCLE database, the Human Protein Atlas, and GEPIA). Most research has been conducted on the choriocarcinoma JAR and JEG3 cell lines. However, choriocarcinoma represents a very rare histological subtype among TGCT patients, especially when present as a pure choriocarcinoma. However, it is considered a very aggressive type of testicular cancer, as the disease outcomes of these patients are very poor [61]. Therefore, it is important to evaluate novel treatment modalities for these patients. To the best of our knowledge, there are virtually no data regarding *FOLR1* expression in choriocarcinomas in the TCGA database, possibly due to its rare occurrence. Intriguingly, our study shows that choriocarcinoma cell lines conveyed the most sensitive responses to MIRV treatment in both 2D and 3D cell cultures. Overall, our findings provide experimental evidence for further assessments targeting FRα in TGCTs, its expression in patient samples, and its association with clinicopathological characteristics. Importantly, our data also demonstrate the efficacy of MIRV in EC cells with moderate/low FRα antigen expression.

ADCs can induce a bystander effect—an ability to mediate cell death indirectly in adjacent tumor cells with low antigen expression. This is a desirable biological activity, tackling the caveats of tumor heterogeneity and improving the antitumor effect [14,32,62]. Previously, the BAT8006 compound—with high affinity for the FRα antigen, composed of an antibody bound to an enzymatically cleavable linker and a topoisomerase I inhibitor as the payload—was found to be a potent inhibitor of FRα-negative cell proliferation through the addition of culture medium from BAT8006-treated FRα-positive cells [22]. We demonstrated the bystander effect of MIRV in direct coculture by assessing the viability of FRα-positive and FRα-negative tumor cells at a 12.5 nM concentration. This is of particular importance and clinical relevance in tumors with mixed histology and heterogeneous antigen expression.

In this study, we observed significantly higher *FOLR1* levels in patients with TGCTs who achieved unfavorable responses to first-line therapy (other than complete remission and/or partial remission with negative serum tumor markers). This observation was most pronounced in the intermediate and poor prognosis subgroups. Moreover, we observed a higher level of *FOLR1* in postchemotherapy-viable tumors than in chemotherapy-naïve testicular germ cell tumors.

## 5. Conclusions

For the first time, we showed significantly higher *FOLR1* levels in patients with TGCTs who achieved an unfavorable response and postchemotherapy-viable tumors compared with chemotherapy-naïve testicular germ cell tumors. These patients’ data suggest *FOLR1* involvement in cisplatin resistance in TGCTs and support targeting *FOLR1* with MIRV in refractory GCTs.

Our data identify treatment-refractory (T)GCTs as clinical entities suitable for salvage therapy with FRα-targeting agents, specifically showing the utility and high efficacy of MIRV for further testing in a preclinical setting.

## Figures and Tables

**Table 1:** Patient characteristics.

	*N*	%
All patients	64	100.0
Histology		
Seminoma	17	26.6
Non-seminoma	47	73.4
Tumor primary		
Testis	64	100.0
Retroperitoneum	0	0.0
Mediastinum	0	0.0
Treatment response		
Favorable	59	92.2
Unfavorable	4	6.3
Response (IA and IB excluded)		
Favorable	51	79.7
Unfavorable	5	7.8
Response risk IGGCCG groups 2 and 3		
Favorable	11	17.2
Unfavorable	5	7.8
S-stage		
S0	23	35.9
S1	23	35.9
S2	10	15.6
S3	8	12.5
IGGCCG risk group		
Good	48	75.0
Intermediate	5	7.8
Poor	11	17.2
Metastases		
Retroperitoneum		
Absent	13	20.3
Present	51	79.7
Mediastinum		
Absent	58	90.6
Present	6	9.4
Other LN		
Absent	52	81.3
Present	12	18.8
Lung		
Absent	49	76.6
Present	15	23.4
Liver		
Absent	57	89.1
Present	7	10.9
Brain		0.0
Absent	64	100.0
Present	NA	
Other mts		
Absent	61	95.3
Present	3	4.7
Number of metastatic sites		
0	11	17.2
1 to 2	41	64.1
3 or more	12	18.8

## Data Availability

The data are available from the corresponding author upon reasonable request.

## Supplementary Materials

The following supporting information can be downloaded at: https://www.mdpi.com/article/10.3390/cells14040287/s1.
